# Differential item functioning (DIF) analyses of health-related quality of life instruments using logistic regression

**DOI:** 10.1186/1477-7525-8-81

**Published:** 2010-08-04

**Authors:** Neil W Scott, Peter M Fayers, Neil K Aaronson, Andrew Bottomley, Alexander de Graeff, Mogens Groenvold, Chad Gundy, Michael Koller, Morten A Petersen, Mirjam AG Sprangers

**Affiliations:** 1 Section of Population Health, University of Aberdeen, UK; 2 Department of Cancer Research and Molecular Medicine, Faculty of Medicine, Norwegian University of Science and Technology, Trondheim, Norway; 3 Division of Psychosocial Research and Epidemiology, Netherlands Cancer Institute, Amsterdam, Netherlands; 4 Quality of Life Department, European Organisation for Research and Treatment of Cancer Headquarters, Brussels, Belgium; 5 Division of Medical Oncology, Department of Internal Medicine, University Medical Centre, Utrecht, Netherlands; 6 Department of Palliative Medicine, Bispebjerg Hospital, Copenhagen, Denmark; 7 Institute of Public Health, University of Copenhagen, Denmark; 8 Centre for Clinical Studies, University Hospital Regensburg, Regensburg, Germany; 9 Department of Medical Psychology, Academic Medical Centre, University of Amsterdam, Netherlands

## Abstract

**Background:**

Differential item functioning (DIF) methods can be used to determine whether different subgroups respond differently to particular items within a health-related quality of life (HRQoL) subscale, after allowing for overall subgroup differences in that scale. This article reviews issues that arise when testing for DIF in HRQoL instruments. We focus on logistic regression methods, which are often used because of their efficiency, simplicity and ease of application.

**Methods:**

A review of logistic regression DIF analyses in HRQoL was undertaken. Methodological articles from other fields and using other DIF methods were also included if considered relevant.

**Results:**

There are many competing approaches for the conduct of DIF analyses and many criteria for determining what constitutes significant DIF. DIF in short scales, as commonly found in HRQL instruments, may be more difficult to interpret. Qualitative methods may aid interpretation of such DIF analyses.

**Conclusions:**

A number of methodological choices must be made when applying logistic regression for DIF analyses, and many of these affect the results. We provide recommendations based on reviewing the current evidence. Although the focus is on logistic regression, many of our results should be applicable to DIF analyses in general. There is a need for more empirical and theoretical work in this area.

## Background

Many health-related quality of life (HRQoL) instruments contain multi-item scales. As part of the process of validating a HRQoL instrument it may be desirable to know whether each item behaves in the same way for different subgroups of respondents. For example, do males and females respond differently to a question about carrying heavy objects, even after accounting for their overall level of physical functioning? Is an item about fatigue answered similarly by older and younger age groups, given the same overall fatigue level? Does a translation of a questionnaire item behave in the same way as the original version? Differential item functioning (DIF) methods are a range of techniques that are increasingly being used to evaluate whether different subgroups respond differently to particular items within a scale, after controlling for group differences in the overall HRQoL domain being assessed.

DIF analyses were first used in educational testing settings to investigate whether particular items in a test were unfair to, for example, females or a particular ethnic group, even after adjusting for that group's overall test ability. In HRQoL research, similar analyses may be used to assess whether there are differences in response to a particular subscale item as a function of respondent characteristics such as age group, gender, education or treatment, given the same level of HRQoL. DIF analyses may also be employed to evaluate cross-cultural response differences, e.g. by country or ethnicity or to evaluate translations of questionnaire items. Whereas in educational settings, items with DIF may simply be dropped or replaced, this may be less straightforward in HRQoL settings if an instrument is already established.

DIF analyses can be carried out using a wide range of statistical methods to explore the relationship between three variables: is group membership (*g*) associated with differential responses (*x*_*i*_) to an item (*x*) for respondents at the same level of a matching criterion (*θ*)? For example, DIF analyses examining the effect of gender on a particular pain item consider not only the proportions of males and females choosing each item category, but also the possibility that males and females report different levels of overall pain as measured by the other pain items.

The **grouping variable **(or **exogenous variable**) *g *may be binary, such as male/female, or may have multiple categories. The item response (*x*_*i*_) may be binary (e.g. yes/no) or ordered categorical (e.g. good/fair/poor). The **matching criterion **or **matching variable **(*θ*) is used to account for different levels of functioning or ability in each group. For some DIF methods, an observed scale score (frequently the sum of the items) is used as the matching variable; in other methods a latent variable is used.

Two distinct types of DIF can be distinguished. **Uniform DIF **occurs if an item shows the same amount of DIF whatever the level of *θ*. When **non-uniform DIF **is present, the magnitude of the effect varies according to *θ*. For example, non-uniform gender DIF might occur in a pain item if it were found that males with lower levels of pain were more likely to score higher on an item compared with female respondents, whereas males with severe pain might be relatively less likely than females to score highly. Detection procedures should attempt to assess both uniform and non-uniform DIF, although in practice not all methods can detect non-uniform DIF.

The literature on DIF is diverse because there is a wide choice of methodologies that may be employed, including contingency table, item response theory (IRT), structural equation modelling and logistic regression methods. Although these represent very different methodological approaches, there are also many challenges that may be encountered regardless of the DIF method used. One widely used approach for detecting DIF is logistic regression, which is commonly regarded as simple, robust and reasonably efficient, while being easy to implement. This paper focuses primarily on the use of the logistic regression method, although many of the conclusions are likely to be equally pertinent to other DIF methods, and is intended to complement existing review articles on logistic regression DIF [1,2], which have a somewhat different focus to our review.

## Aim

The specific aim of this article is to provide an overview of the logistic regression approach to DIF detection. The review also considers more general methodological issues specific to DIF analyses of HRQoL instruments, including the evaluation of DIF in short scales and the problems with interpreting DIF.

## Methods

Although this should not be considered a systematic review as judgement was used to select included articles, a systematic search strategy using the search term "differential item functioning" was employed to identify relevant articles using the electronic databases MEDLINE, EMBASE and Web of Knowledge. Abstracts of the articles were assessed for relevance and a decision made whether or not to review the full article. Priority was given to studies concerning HRQoL instruments, but as DIF analyses originated in educational testing, much of the literature relates to educational settings. DIF studies from other areas were therefore included if considered to have broader methodological relevance. Although the greatest emphasis was placed on articles using logistic regression techniques, articles relating to any DIF methodology were included if considered relevant to the discussion of specific issues or topics. The electronic literature search was supplemented by relevant articles and books from the reference lists of studies already included.

## Results

A total of 211 (MEDLINE), 147 (EMBASE) and 589 (Web of Knowledge) articles met the initial search criteria. The full text of 136 articles was accessed as part of the review.

DIF detection studies were identified for HRQoL instruments from many clinical areas including: asthma [3], oncology [4-9], headache [10,11], mental health [12-18] and functional ability [19-21].

A wide range of grouping factors has been evaluated in HRQoL DIF studies including: language/translation [7,8,11,12,22], language group [23], country [5,16,19,21,22,24,25], gender [3,10,13,14,17,19,22,25-30], age [4,10,22,25,27,29,30], ethnicity [6,13,15,27,29-31], education [10,28,29], employment status [10], job category [32], treatment [4] and type of condition [22,20].

## Methods for Investigating DIF

A large number of diverse statistical methods for detecting DIF have been described in the literature [33-38]. DIF methods may be divided into parametric methods, requiring distributional assumptions of a particular model, and non-parametric methods that are distribution-free. Provided that the assumptions are met, parametric approaches may be more powerful and stable [37].

Many DIF detection studies have used methods based on item response theory (IRT) [35,39], including a number of recent studies of HRQoL instruments [5,6,20,40]. The main advantage of IRT DIF techniques is the use of a latent (rather than an observed) variable for *θ*, the matching criterion. Disadvantages include possible lack of model fit, increased sample size requirements and the need for more specialised computer software [41].

Contingency table methods, particularly the Mantel-Haenszel and standardisation approaches, are non-parametric methods that are frequently used in educational testing [42,43]. These methods are straightforward to perform and do not require any model assumptions to be satisfied, but are unable to detect non-uniform DIF. These methods have been infrequently used in HRQoL research, although an approach using the partial gamma statistic has been used [36]. Other DIF detection methods include the simultaneous item bias test (SIBTEST) method [44] and approaches using structural equation modelling [45].

### Logistic regression

The remainder of this review will concentrate on the method of logistic regression [1,2,46-49].

For items with two response categories, binary logistic regression can be used to relate the probability of positive response (*p*) to the grouping variable (*g*), the total scale score (representing ability level/level of quality of life) (*θ*) and the interaction of the group and scale score (the product of *g *and *θ*). In HRQoL research, items frequently have three or more ordered response categories, necessitating use of ordinal logistic regression instead. This estimates a single common odds ratio assuming that the odds are proportional across all categories [50].

The binary and ordinal logistic regression models can be written respectively as:

$$\begin{array}{l} \ln\left[\frac{p}{1-p}\right]=\beta_{0}+\beta_{1}\theta +\beta_{2}g+\beta_{3}g\theta \\ \ln\left[\frac{\mathrm{Pr}(Y\leq k|g,\theta )}{1-\mathrm{Pr}(Y\leq k|g,\theta )}\right]=\beta_{0k}+\beta_{1}\theta +\beta_{2}g+\beta_{3}g\theta \;\;\left(k=0,1,2,...\right) \end{array}$$

where Pr(Y ≤ *k*) is the probability of response in category *k *or below (*k *= 0,1,2,...) and *β*_*0k*_*,β*_*1*_*,β*_*2*_, *β*_*3 *_are constants usually estimated by maximum likelihood.

An advantage of logistic regression methods is the ability to test for both uniform and non-uniform DIF. The presence of uniform DIF is evaluated by testing whether the regression coefficient of group membership (*β*_*2*_) differs significantly from zero. A test of the interaction coefficient between group membership and ability (*β*_*3*_) can be used to assess non-uniform DIF.

Some authors advocate first testing the presence of both uniform and non-uniform DIF simultaneously using a test of the null hypothesis that β_2 _= β_3 _= 0 [2,46,47]. The difference in the -2 Log Likelihood (-2LL) of these models is assessed using a chi-squared distribution with two degrees of freedom (2 df). If this step gives a significant result, the presence of uniform DIF alone is then determined by testing the significance of β_2 _using a chi-squared distribution with one degree of freedom (1 df). An alternative strategy is to report two separate 1 df chi-squared tests for uniform and non-uniform DIF [51]. Simulations have shown that this approach may lead to improved performance [49,52].

Perhaps the main advantage of the logistic regression DIF approach is its flexibility [2,53]. For example, if more than two groups are to be compared, extra variables may be included in the regression model to indicate the effect of each group with respect to a reference category. Another advantage is the ease of adjusting for additional covariates, both continuous and categorical, which may confound the DIF analyses. Despite this much-cited benefit, few logistic regression DIF studies making use of adjusted analyses were identified [8]. In fact, given interpretation difficulties, some authors prefer to test each covariate for DIF in separate models [54].

## Methodological issues with DIF Analyses

### Sample size

There are no established guidelines on the sample size required for DIF analyses. The minimum number of respondents will depend on the type of method used, the distribution of the item responses in the two groups, and whether there are equal numbers in each group. For binary logistic regression it has been found that 200 per group is adequate [1], and a sample size of 100 per group has also been reported to be acceptable for items without skewness [55]. For ordinal logistic regression, simulations suggested that 200 per group may be adequate, except for two-item scales [56]. As a general rule of thumb, we suggest a minimum of 200 respondents per group as a requirement for logistic regression DIF analyses.

### Unidimensionality

DIF analyses assume that the underlying distribution of *θ *is unidimensional [34], with all items measuring a single concept; in fact, some authors suggest that DIF is itself a form of multidimensionality [38]. Although it has been recommended that factor analysis methods be used to confirm unidimensionality prior to performing DIF analyses [38], in practice few DIF studies have reported dimensionality analyses [57]. When the construct validation of a HRQoL instrument has already explored scale dimensionality, further testing may be deemed unnecessary.

### Deriving the matching criterion

It might seem counter-intuitive to include the studied item itself when calculating a scale score for the matching criterion, but studies have found that DIF detection was more accurate when this is done [35,58]. Thus, if the matching criterion is the summated scale score, the item being studied should not be excluded from the summation.

### Purification

An item with DIF might bias the scale score estimate, making it less valid as a matching criterion for other items. Some DIF studies have employed "purification" [35], which is an iterative process of eliminating items with the most severe DIF from the matching criterion when assessing other items. Purification has been shown to be beneficial in DIF analyses in other fields [59,60], but has rarely been used in HRQoL research [61], perhaps owing to the lower number of items in HRQoL subscales. We recommend that more consideration be given to purification, although the benefit may depend on the number of items in the scale: it may be less suitable for scales with just a small number of items, as removing items can affect the precision of the matching variable. For these scales, we would recommend more qualitative approaches that attempt to understand underlying reasons for DIF.

### Sum scoring versus IRT scoring

An important disadvantage of the logistic regression method is reliance on an observed scale score, which may not be an adequate matching variable, particularly for short scales [53,62]. Thus, it has been suggested that item response theory (IRT) scoring should be used to derive the matching variable, even when IRT is not itself used for DIF detection. This hybrid logistic regression/IRT method has been used in a number of recent studies and free software is available for this purpose [2,62,63]. It also has the advantage of incorporating purification by using an iterative approach that can account for DIF in other items [63,64]. It is our view, however, that the standard logistic regression approach using sum scores is an acceptable method in practice; reported results of DIF analyses using the hybrid method have tended to be similar to those obtained using sum scores [2].

### Pseudo-DIF

"Pseudo-DIF" results when DIF in one item causes apparent opposing DIF in other items in the same scale, even though these other items are not biased [36]. For example, in logistic regression DIF analyses the log odds ratios for items in a scale will sum approximately to zero. Thus log odds ratios for items without real DIF may be forced into the opposite direction to compensate for items with true DIF. The most extreme case occurs for two-item scales where opposite DIF effects will be found for the two items; the results are therefore impossible to interpret without additional external information (see the section on qualitative methods below) [65].

### Scale length and floor/ceiling effects

In HRQoL research the number of items per scale may vary, and subscales may often contain only a few items in order to minimise the burden on patients. DIF analyses of short scales may be difficult to interpret because of pseudo-DIF and the scale score may also be a less accurate measure of the underlying construct. Several studies have successfully conducted DIF analyses in scales with fewer than ten items [3-5,7-9,11,19,20,22,24,61].

Another common problem with HRQoL instruments is items with floor and ceiling effects, or with highly skewed score distributions. These items will not be able to discriminate between groups as effectively as other items [35,37]. Simulations show that there is reduced power to detect DIF in such items, although Type I error rates appear to be stable [56].

## Interpretation of DIF Analyses

Like many other DIF detection methods, logistic regression uses statistical hypothesis tests to identify DIF. Interpretation of an item with statistically significant DIF is rarely straightforward. It could have arisen purely by chance, it could result from pseudo-DIF in another item in the same scale, or it could be caused by confounding [7,36]. If real DIF does exist there might be more than one possible cause. For example, for DIF analyses of a questionnaire with respect to country, observed DIF could either be caused by a lack of translation equivalence or by cross-cultural response differences. Sample size also affects interpretation of DIF - sufficiently large sample sizes may result in the detection of unimportant yet statistically significant DIF.

### Methods of adjustment for multiple testing

Multiple hypothesis testing may be a particular problem in DIF analyses: there may be more than one HRQoL subscale of interest, analyses may be performed for all items within the scales, and for each item there may be several grouping variables. If some of these grouping variables have several categories (e.g. the translation used), this may involve several tests for each variable. Finally, tests for both uniform and non-uniform DIF may be conducted. The large number of significance tests increases the probability of obtaining false statistically significant results by chance alone.

Multiple testing is common to many statistical applications and the various approaches to address these issues are reviewed elsewhere [66]. One solution is to use a Bonferroni approach (dividing the nominal statistical significance level, typically 0.05, by the number of tests conducted); this reduces the Type I errors, but is a very conservative approach. Some DIF studies have used a 1% significance level instead [19,55,67]. An alternative approach is to use cross-validation, whereby the data are randomly divided into two datasets, and one of the halves is used to confirm the results obtained on the other half [4,24]. In general, researchers investigating DIF should account for the number of significance tests conducted, unless they regard the search for DIF as hypothesis-generating and report their findings as tentative, in which case multiple testing is arguably less of an issue [62].

### Methods of determining clinical significance

Since statistical significance does not necessarily imply clinical or practical significance, many authors have proposed DIF classifications that incorporate both statistical significance and the magnitude of DIF, but once again the question of which thresholds to use is not straightforward.

One widely used approach is first to calculate statistical significance using the standard likelihood ratio test and then to calculate, as a measure of effect size, the change in the R^2 ^associated with including the grouping variable in the model. For ordinal logistic regression a measure such as McKelvey and Zavoina's pseudo-R^2 ^may be used [1]. Non-uniform DIF may be assessed similarly [68].

Two sets of rules have been developed to classify DIF using the change in R^2^, the Zumbo-Thomas procedure [1] and the Jodoin-Gierl approach [49]. The corresponding cut-offs for indicating moderate and large DIF are very different: 0.13 and 0.26 for Zumbo-Thomas and 0.035 and 0.070 for Jodoin-Gierl. Both systems usually require a p-value of less than 0.001. Unsurprisingly, these criteria can produce very different numbers of items flagged with DIF [49,69] and several authors have also remarked that Zumbo's method is very conservative and that few items meet the criteria [23,55]. An R^2 ^difference cut-off level of 0.02 has also been suggested by Bjorner et al. (2003), and used in other studies [10,11,22,25], whereas Kristensen et al. (2004) used a rule that the group variable had to explain at least 5% of the item variation after adjusting for the sum score [32].

Crane has suggested testing for non-uniform DIF using a Bonferroni-corrected likelihood ratio chi-squared test with 1 df. For uniform DIF, significance criteria are not used: the change in the regression coefficient for *θ *in models with and without the group variable is calculated and a 10% difference is used to indicate important DIF [2,62]. In a more recent study, a 5% difference was used [63].

In logistic regression DIF analyses, the odds ratio associated with the grouping variable can also be used as a magnitude criterion. For example, Cole et al. (2000) used proportional odds ratios greater than 2 or less than 0.5 to denote practically meaningful DIF [27]. A classification system adapted from that used in educational testing has also been used with odds ratios [70]. Slight to moderate DIF is indicated by a statistically significant odds ratio that is also outside the interval 0.65 to 1.53; moderate to large DIF is indicated if the odds ratio is outside 0.53 to 1.89 and significantly less than 0.65 or greater than 1.53 [24]. A number of studies have used a threshold in the log odds ratios of 0.64 (≈ln(0.53)), often in conjunction with p < 0.001 [7-9,61].

A recent study compared three assessment criteria for evaluating two composite scales formed from items taken from a number of HRQoL instruments [71]: Swaminathan and Roger's approach using only statistical significance [46], Zumbo and Gelin's pseudo-R^2 ^magnitude criterion [14], and Crane's 5% change in the regression coefficient [2]. The three methods flagged very different numbers of items as having DIF. This is not surprising and stems partly from the dichotomisation of DIF effects into either DIF or no DIF, when in fact it is a matter of degree [72]. There is currently no consensus regarding effect size classification system for logistic regression DIF analyses, and there is a need for further investigation [49]. What is of primary importance is that results of the statistical significance tests should not be interpreted without reference to their clinical significance.

### Illustration of DIF

Some authors advocate the use of graphical methods to display the magnitude and direction of DIF effects [73]. Forest plots may provide a convenient way to summarise the pattern of DIF across several categories [8]. Crane's logistic regression software produces box and whisker plots to evaluate the impact of DIF on each covariate [63,74,75].

### What should be done if DIF is found?

Unfortunately, the DIF literature tends to focus on how to detect DIF, rather than on what to do when it is found, but there are two main steps that may be employed. First, if significant DIF, uniform or non-uniform, is found, detailed examination of the three-way contingency table of item, scale score and grouping variable can help interpret the direction and nature of this DIF effect. It may then be helpful to identify underlying reasons for the differential functioning using expert item review (see the section on qualitative methods below).

The second approach is to determine the practical impact of observed DIF. This can be assessed, for example, by removing items with DIF and determining what difference this makes to the results [76]. Impact analyses have also been used to investigate whether item-level DIF results in clinically important differences at the scale level [77]. Some authors have attempted to use IRT methods to adjust their results and correct for the presence of DIF [6,7,63]. Others have argued that at the scale level DIF due to multidimensionality may in fact balance out [78].

If an instrument is at the development stage, modifications can also be made to items before retesting in further DIF analyses. If translation DIF is found for a particular item, the wording may be reviewed by independent translators. It becomes more problematic when a DIF effect is found for an established HRQoL questionnaire: researchers need to consider carefully how this will affect future studies. For example, if DIF is found with respect to age group, this may not be important for a study with narrow age inclusion criteria, but it would be for studies including both older and younger participants. DIF may also have lower impact on clinical trials than on observational studies as randomisation may ensure groups are balanced with respect to important patient characteristics [77].

### Use of qualitative methods alongside DIF analyses

Some authors have attempted to interpret the underlying causes of flagged DIF, either anecdotally or by using formal qualitative methods. Studies in the educational field have, however, typically found low agreement between expert reviews of items and statistical DIF analyses [34,57]. For example, many HRQoL instruments are translated into other languages or undergo cultural adaptation for use in another country. DIF analyses may be useful for evaluating item translations and, if DIF is found, the relevant wording may be reviewed. It may be difficult, however, to separate lack of translation equivalence from cross-cultural response differences.

We identified only a few studies that attempted to relate DIF results to blinded substantive assessments of the reasons for DIF: most conducted in fields such as educational testing [8,67,79-85]. A number of studies attempted to give post hoc explanations for DIF effects found in HRQoL instruments [4,6,7,12,16,19,22,24,25,86]. Where resources exist to do this, we recommend that researchers employ expert review of DIF items as part of the process of understanding and interpreting DIF effects. They are particularly useful in situations with more than one possible source of DIF, such as when distinguishing between cultural and linguistic response differences in DIF analyses of translations. A more detailed review of the studies using external information alongside DIF analyses may be found elsewhere [65].

## Summary

Although much of the published research on DIF methods concerns educational tests, DIF techniques are increasingly being applied to HRQoL outcomes. This introduces a new set of challenges. HRQoL scales often consist of short scales with ordered categorical items, and some items may exhibit floor and ceiling effects. Pseudo-DIF may be a problem, and without parallel qualitative methods the underlying causes of the DIF effects may not be clear.

Many methods for DIF detection are available, and this review has focused largely on just one such approach: logistic regression. This method has several advantages in the context of HRQoL DIF analyses, but a disadvantage is the reliance on sum scores as the matching variable. IRT DIF methods using a latent matching variable have important theoretical advantages but these may be less accessible to those with only standard statistical software. The hybrid logistic regression/IRT method has been employed successfully in several studies although the evidence of tangible practical benefit over the standard sum score method is limited.

There are many competing criteria for determining what constitutes important DIF, using either statistical significance or magnitude criteria, and these have been shown to flag different numbers of items with DIF. In educational contexts the level of DIF that is important is a matter of policy, and practical considerations are most important [35]. Similarly, although DIF analysis is an important tool in HRQoL research, it cannot be employed on its own: judgement should be used alongside the statistical results when deciding whether a particular DIF effect is of sufficient practical importance to require modification of an item or scale.

The choices made during analysis will substantially affect the results, and we have described and illustrated the impact of these choices. We have reviewed the literature and provided guidance for making the decisions about the optimal application of logistic regression for DIF analysis. Many of these findings are likely to be equally pertinent to other approaches for detecting DIF.

## Key Messages

• A variety of DIF methodologies are available. For HRQoL instruments, logistic regression is a robust and flexible method and therefore a good practical choice in most situations. A hybrid logistic regression/IRT method, which avoids the theoretical disadvantages of using the sum score as a matching variable, is also available.

• A combination of statistical significance and magnitude criteria should be used when classifying items as having DIF. When interpreting results, allowance should be made for the number of tests conducted.

• When deriving the matching criterion for logistic regression DIF using sum scores, the overall scale score including the studied item should be used.

• For longer scales researchers should consider iteratively eliminating items with DIF in subsequent DIF analyses (purification).

• Prior to conducting DIF analyses, it should be checked that a scale is unidimensional.

• At least 200 respondents per group are recommended for logistic regression DIF analyses.

• Graphical methods may be used to display DIF results in multiple groups.

## Competing interests

The authors declare that they have no competing interests.

## Authors' contributions

NWS conducted the literature review and wrote the first draft of the article. PMF, NKA, AB, AdG, MG, CG, MK, MAP and MAGS contributed to subsequent drafts. All authors read and approved the final version.
